# The psychosis treatment gap and its consequences in rural Ethiopia

**DOI:** 10.1186/s12888-019-2281-6

**Published:** 2019-10-29

**Authors:** Abebaw Fekadu, Girmay Medhin, Crick Lund, Mary DeSilva, Medhin Selamu, Atalay Alem, Laura Asher, Rahel Birhane, Vikram Patel, Maji Hailemariam, Teshome Shibre, Graham Thornicroft, Martin Prince, Charlotte Hanlon

**Affiliations:** 1 Center for Innovative Drug Development and Therapeutic Trials for Africa (CDT-Africa), Addis Ababa University, Addis Ababa, Ethiopia; 2 Global Health & Infection Department, Brighton and Sussex Medical School, Brighton, UK; 3 College of Health Sciences, School of Medicine, Department of Psychiatry, Addis Ababa University, Addis Ababa, Ethiopia; 4 Aklilu Lemma Institute of Pathobiology, Addis Ababa University, Addis Ababa, Ethiopia; 5 Alan J Flisher Centre for Public Mental Health, Department of Psychiatry and Mental Health, Addis Ababa University, University of Cape Town, Cape Town, South Africa; 6 Centre for Global Mental Health, Health Service and Population Research Department, King’s College London, Institute of Psychiatry, Psychology and Neuroscience, London, UK; 7 The Wellcome Trust, London, UK; 8 Division of Epidemiology and Public Health, School of Medicine, University of Nottingham, Nottingham, UK; 9 Department of Global Health & Social Medicine, Harvard Medical School, Boston, USA; 10 Department of Psychiatry, Dalhousie University, Horizon Zone 3, Fredericton, NB Canada

**Keywords:** Treatment gap, The Butajira treatment gap questionnaire, Treatment coverage, Treatment access, Severe mental disorder, Low and middle-income country, Developing country

## Abstract

**Background:**

The “treatment gap” (TG) for mental disorders, widely advocated by the WHO in low-and middle-income countries, is an important indicator of the extent to which a health system fails to meet the care needs of people with mental disorder at the population level. While there is limited research on the TG in these countries, there is even a greater paucity of studies looking at TG beyond a unidimensional understanding. This study explores several dimensions of the TG construct for people with psychosis in Sodo, a rural district in Ethiopia, and its implications for building a more holistic capacity for mental health services.

**Method:**

The study was a cross-sectional survey of 300 adult participants with psychosis identified through community-based case detection and confirmed through subsequent structured clinical evaluations. The Butajira Treatment Gap Questionnaire (TGQ), a new customised tool with 83 items developed by the Ethiopia research team, was administered to evaluate several TG dimensions (access, adequacy and effectiveness of treatment, and impact/consequence of the treatment gap) across a range of provider types corresponding with the WHO pyramid service framework.

**Results:**

Lifetime and current access gap for biomedical care were 41.8 and 59.9% respectively while the corresponding figures for faith and traditional healing (FTH) were 15.1 and 45.2%. Of those who had received biomedical care for their current episode, 71.7% did not receive minimally adequate care. Support from the community and non-governmental organisations (NGOs) were negligible. Those with education (Adj. OR: 2.1; 95% CI: 1.2, 3.8) and history of use of FTH (Adj. OR: 3.2; 95% CI: 1.9–5.4) were more likely to use biomedical care. Inadequate biomedical care was associated with increased lifetime risk of adverse experiences, such as history of restraint, homelessness, accidents and assaults.

**Conclusion:**

This is the first study of its kind. Viewing TG not as a unidimensional, but as a complex, multi-dimensional construct, offers a more realistic and holistic understanding of health beliefs, help-seeking behaviors, and need for care. The reconceptualized multidimensional TG construct could assist mental health services capacity building advocacy and policy efforts and allow community and NGOs play a larger role in supporting mental healthcare.

## Background

The treatment gap is an important concept in global health advocacy with applicability across a range of chronic medical conditions such as HIV/AIDS [1], hypertension [2], cardiovascular diseases [3], diabetes [3], epilepsy [4] and mental disorders [5]. For all conditions, the treatment gap is defined as the proportion of people with disorder who require an intervention but do not receive one. The treatment gap for mental disorders is universally large, although particularly marked in low and middle-income countries (LMIC) [5, 6], with almost four out of five persons with severe mental disorders in LMIC receiving no treatment in the previous year [7, 8]. This is even larger in sub-Saharan Africa with nine in ten people with schizophrenia not receiving care [9, 10]. In Ethiopia, the Butajira study on the course and outcome of severe mental illnesses 15 years ago, reported a lifetime treatment access gap for schizophrenia and bipolar disorder of 90% [11, 12], with similar national rates more recently [9].

The treatment gap is an indicator of the extent to which a health system fails to meet the care needs of people with a specific disorder at the population level. As such, changes in the treatment gap is an important metric for tracking progress in improving treatment coverage in moving towards universal health care [13]. However, current measures of the treatment gap, consisting of direct and indirect approaches, are conceptually inadequate and are criticised for ignoring the broader range of services or ‘plurality’ of care [14]. The potential negative consequences of not receiving care, particularly relevant in places with high ‘treatment gap’, where potential for human rights violations may be substantial [15], are also overlooked. Thus, broadening the definition and applicability of the treatment gap to varied contexts, interventions and outcomes is pertinent. In this paper we re-conceptualise the treatment gap as a multi-dimensional construct and evaluate its burden in people with psychosis at the point of engagement with a new integrated service in rural Ethiopia.

### Re-conceptualising the treatment gap

**Fig. 1:**
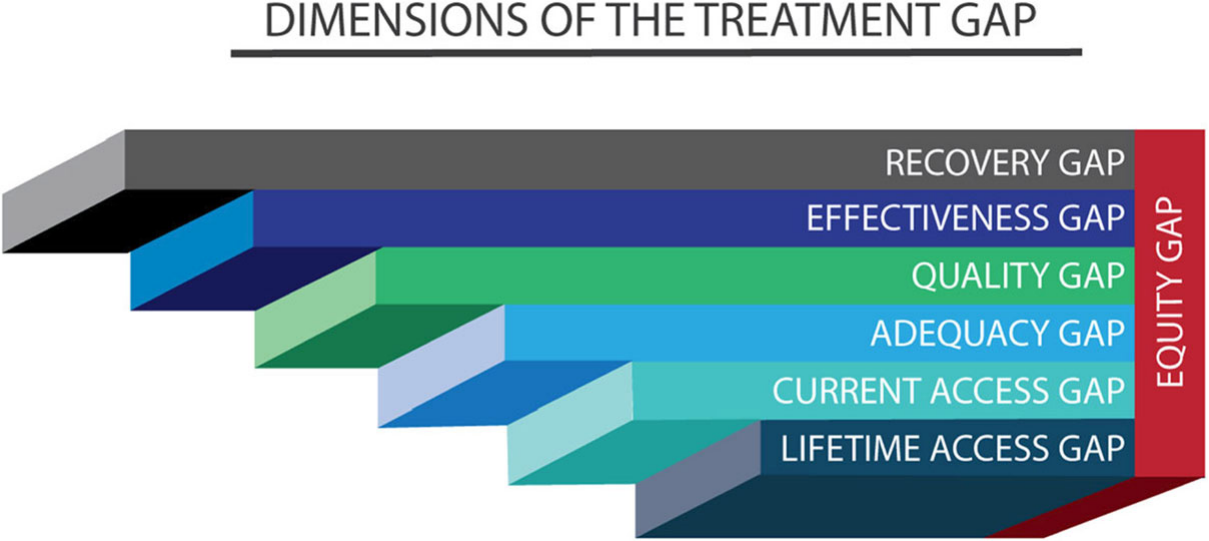
Dimensions of the treatment gap continuum. It is hypothesized that lifetime access gap would be the smallest, while recovery gap would be the largest. Equity (whether access to adequate, quality and effective treatment provision is affected by various personal and demographic characteristics) is relevant to all the treatment gap types

Our re-conceptualisation is based on two premises. First, as indicated above, is the need to consider the plurality of care and the power of individuals to use the care they choose. The service pyramid of the WHO [16] is a useful framework for defining and measuring this plurality of care. In addition to biomedical care, it is contextually appropriate to quantify access to FTH providers as well as support from the community, non-governmental organisations, family and self-care. The second premise is the need to move away from treatment for a disorder to the goal of treatment, “recovery” and “recovery” gap with emphasis on what is meaningful to the person in need. In this regard, the treatment gap is viewed as a continuum, with the continuum moving from lack of access to any evidence-based care during the whole duration of the illness (lifetime access gap) to failure to achieve the goal of treatment, recovery (recovery gap) (Table 1 and Fig. 1). The most severe form of access gap is the *lifetime access gap*, which provides information about the severity of population level neglect, and may have particular relevance in LMIC settings.

**Table 1:** Definitions of the treatment gap dimensions and how they may be measured

Care/treatment gap dimensions	Definition	How measured	
		Subjective	Objective
Access			
• Lifetime	Whether there ‘ever’ was access to evidence-based care since onset of illness without any judgment about efficacy	Self-reported access over the course of illness since onset	Linkage based on databases (electronic or other records)
• Current	Whether there was access to evidence-based care for the current or most recent episode of illness	Self-reported access during the current or most recent episode of illness	Linkage based on databases (electronic or other records)
Adequacy	Whether adequate quantity of treatment was provided in terms of the nature, dose and duration of treatment	Self-reported minimum adequacy standard	Recorded information compared with established standard of care
Quality	Attainment of a certain standard and meeting certain intrinsic characteristics of care such as patient satisfaction and concordance with patient values	Self-reported patient satisfaction	Evaluation of whether care is concordant with established quality standards and guidelines
Effectiveness	Intended outcomes of clinical improvement achieved with little untoward consequences and inconvenience to user	Self-reported benefit of care	Standard scales of effectiveness
Recovery	This is the ultimate goal of treatment and understood in three ways: • Sustained clinical wellness (well for at least 6 months) • Functional wellness (regaining full functionality) • As a process of change that allows individuals to “improve their health and wellness, live a self-directed life, and strive to reach their full potential” [17]	Self-reported recovery	Standard scales of recovery may be used
Equity	Is relevant to all dimensions of care or treatment gap and equitable care ensures that access, quality or impact of care “does not vary in quality because of personal characteristics such as gender, race, ethnicity, geographical location, or socioeconomic status.”		Analysis of variation of care and treatment gap by the various equity dimensions.

The *quality and adequacy gaps* are directly relevant to effectiveness and recovery gaps. Although ‘quality’ has several meanings in health service research, the quality gap here represents how different the care provided is to accepted quality standards or treatment guidelines and to implicit requirements such as patient satisfaction [18].

The *adequacy gap* relates to the adequacy of treatment in terms of dose/intensity, continuity and duration. A simple method of measuring the adequacy gap may be assessing the frequency of service encounters in combination with the appropriateness of the prescribed treatment [19]. Ultimately, the goal of treatment is to achieve full recovery [20]; thus, the target goal for policy initiatives and care provision has to be to reduce the recovery gap. The recovery gap is an important indicator of the inadequacies of the implementation of current evidence-based care. For example, a large proportion of patients receiving treatment for severe [21] or less severe illnesses [22] fail to achieve recovery.

There are two additional dimensions, which are of major importance: equity and impact or consequence. *Equity* is a cross-cutting dimension and a reflection of whether the lack of treatment or the benefits of treatment are distributed across the whole population in need without discrimination. The final dimension of the treatment gap evaluates the *consequence* or impact of the treatment gap on the affected individual, family and the wider community. Estimating the consequences of the treatment gap will show why the treatment gap matters. In addition to the direct illness burden, one of the key consequences of the treatment gap is human rights abuse from various sources including through the process of receiving care.

Redefining the treatment gap in this more nuanced multi-dimensional way extends applicability to ore settings and allows for a more refined analysis and identification of targeted policy interventions.

The aim of this study was to determine the various dimensions of the treatment gap for psychosis in a setting where a new service programme, the Programme for Improving Mental Healthcare (PRIME) [23], was being implemented.

## Methods

The study was a cross-sectional assessment of adults with confirmed diagnosis of psychosis. The study participants were identified through community case detection and subsequent structured clinical evaluation of diagnosis.

### Setting

The study was conducted in the Sodo district, Gurage Zone, Southern Nations, Nationalities and Peoples’ Region (SNNPR) of Ethiopia. We have reported previously on the study setting [24, 25]. Sodo is a predominantly rural district located about 100 km south of the capital city, Addis Ababa. The district hosts one primary hospital, eight health centres and 58 health posts (community based health facilities).

### Case identification

**Fig. 2:**
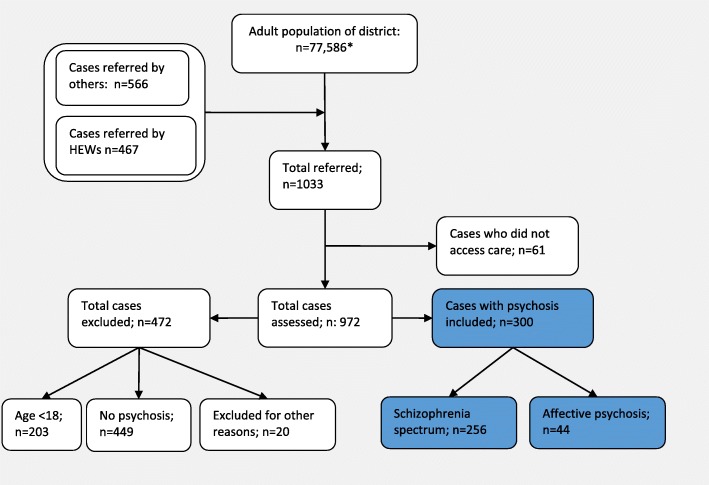
Flow diagram of patient recruitment (*Assuming 54% of the total population to be adults)

We used a two-stage case identification process for recruiting participants (Fig. 2).

First, potential cases with psychosis were identified and referred by community key informants [26], consisting of health extension workers and community leaders trained for half a day by a psychiatrist with experience in training key informants. Health extension workers are healthcare staff with one year of training in healthcare after completing high school education. They staff the health posts located within the communities and also reside within the communities they serve. These health workers visit households about once a month and have intimate knowledge of their communities. Second, these potential cases were referred to the health centres where trained psychiatric nurses conducted a semi-structured interview to confirm diagnosis and evaluate other clinical parameters, such as symptom severity. To be included in the study, participants had to be at least 18 years of age, fulfil diagnostic criteria of the International Classification of Diseases (ICD) [27] for one of the major psychotic disorders ((ICD-10 F20 and ICD-10 F30 [psychotic subsections]), be in need of mental health care at the time of detection, and were resident in the area for at least six months. The study was conducted between December 2014 and August 2015.

### Assessment of diagnosis and other clinical and social parameters

The Operational Criteria for Research (OPCRIT) [28], a semi-structured checklist for genetic studies, was used to support clinical diagnosis. The instrument uses some of the rating styles of the Schedules for Clinical Assessment in Neuropsychiatry (SCAN) [29] but is briefer and simpler to administer. It has established reliability and allows application of multiple diagnostic criteria [28].

### Measurement of the treatment gap

The Butajira Treatment Gap Questionnaire (TGQ) was used to establish the treatment gap (available at http://bit.ly/2oPlqmQ). The TGQ is an 83 items questionnaire exploring receipt of: (1) biomedical care; (2) Faith and Traditional Healing (FTH); (3) Community care (assistance from community residents and leaders, religious institutions, social organisations, NGOs); (4) support from family and friends; (5) general self-care; (6) overall experience and impact or consequence of treatment gap and dignity in care. Details within these main dimensions explored four treatment gap themes or dimensions (Fig. 1): Access to care (lifetime and current access); adequacy of care (for the current access); quality of care (for the current access); and effectiveness of care (perceived benefit of care for the current access).

Adequacy of care was adapted from a study by Wang and colleagues that used frequency of visits as an indicator of adequacy [19]. Thus, based on evidence from primary and speciality care, Wang and colleagues considered four or more visits of follow-up and medication monitoring for “acute and continuation phases of treatment for mood, anxiety and psychotic disorders” as minimally adequate. Quality of care was assessed through satisfaction with provided care. Effectiveness was measured from the participants’ perspective, in terms of whether they felt they had benefited from or harmed by the treatment they received. Under the FTH section, 12 types of locally relevant “healing” providers were included. The most widely used FTH across the country is “Holy Water” treatment, in which water which has been sanctified through prayer is sprinkled on a patient for healing and protection. Finally, in a section on “dignity in care”, the overall experience of care was assessed with a focus on negative experiences, including homelessness, accidents and assaults, restraint and imprisonment. The questions to estimate the treatment gap assessed positive care receipt from which the treatment gap was estimated.

The TGQ was developed as a pragmatic field tool by the Ethiopia team through a series of consensus meetings to agree on the key dimensions of the TG and how to measure these dimensions. The study was part of an initial pilot of the tool. We have not carried out formal validation study. Nevertheless, the reliability of the scale measured through the internal consistency coefficients, Cronbach’s alpha, was generally satisfactory—highest score was obtained for perceived benefit in care or recovery (α = 0.97). The coefficient for quality of care was also good (α = 0.83).

### Illness severity, and other measures

Clinical severity of symptoms was assessed with the Brief Psychiatric Rating Scale- Expanded version (BPRS- E) [30], a 24-item instrument, which has been used previously in Ethiopia [31]. The World Health Organization Disability Assessment Schedule (WHODAS 2.0, [32]), which measures the level of difficulty in daily activities and social participation experienced in the previous 30 days [33] and has been adapted for use in Ethiopia [34, 35] was employed to measure functional impairment. The quality of social support was assessed with the Oslo 3 Social Support Scale (OSS) [36].

### Administration of assessment instruments

The main clinical assessment instruments (OPCRIT and BPRS-E) were administered by trained psychiatric nurses, while the TGQ and the other psychosocial scales were administered by trained lay data collectors. These data collectors were high school graduates with two to four years of additional technical or professional training. They were trained for five days for the data collection and by the time they administered these instruments they already had a one year experience of administering various instruments for the PRIME study.

### Data management

Data were double-entered into Epidata version 3.1 and analysed using STATA version 13.1 (StataCorp, 1985–2013). Simple descriptive analyses were used to summarise socio-demographic factors along with service use and treatment gap profiles.

An exploratory multivariable analysis was carried out using logistic regression to assess for factors associated with the use of biomedical services in the current access. The selected factors were considered theoretically relevant determinants of use of services, such as education, income, social support and service use behaviour as indicated by the use of FTH. Further exploratory analysis included evaluation of the potential link between adequacy of biomedical care and adverse experiences.

## Results

### Demographic and clinical characteristics

A total of 300 participants were included in the study. Participants were predominantly of the Gurage ethnic origin (*n* = 285; 94.7%), Orthodox Christian (*n* = 271; 90.0%) and rural residents (*n* = 240; 80%). Men were slightly overrepresented (*n* = 173; 57.5%) (Table 2). Over four fifths had a diagnosis of a schizophrenia spectrum disorder (*n* = 244; 81.3%) (Table 2). A small minority had affective psychosis (*n* = 40; 13.3%). Overall, participants had a moderate severity of illness and disability measured with the BPRS-E (mean, SD = 47.3, 17.1) and WHODAS (mean, SD = 51.5, 23.5).

**Table 2:** Background characteristics of participants (*n* = 300 unless specified)

Characteristics		Number	Percent
Gender	Male	172	57.3
	Female	128	42.7
Age	18–24	65	21.7
	25–34	82	27.3
	35–44	79	26.3
	45–54	46	15.3
	55 and above	28	9.3
Residence	Urban	60	20.1
	Rural	239	79.9
Education	Illiterate	118	39.3
	No formal education but can read and write	39	13.0
	Formal education	143	47.7
Employment (*n* = 299)	Agricultural work	76	25.4
	self employed	16	5.4
	House wife	58	19.4
	Other employment	39	13.0
	Unemployed	110	36.8
Income	low and below	191	.63.7
	Medium and above	109	36.3
Marital status	Single	136	45.3
	Married	111	37.0
	Divorced	40	13.3
	Widowed	13	4.3
Religion	Orthodox Christian	271	90.0
	Other	30	10.0
Ethnicity (n = 299)	Gurage	281	94.0
	Other	18	6.0
Children (*n* = 295)	Yes	157	53.2
	No	138	46.8
Children under 18 (*n* = 157)	Yes	126	80.3
Summary diagnosis	Schizophrenia spectrum disorders	256	85.3
	Affective psychosis	44	14.7

### The treatment gap

#### Lifetime access gap

The lifetime access to FTH was the highest (Table 3), with 84.9% (*n* = 254) of participants having accessed this modality of care. Over half of the participants (58.2% (*n* = 174) had accessed biomedical care (specialist mental health services) at some point during the illness. Thus, the lifetime access gap was 15 and 41.8% for FTH and biomedical care respectively. Lifetime experience of admission (staying for at least 24 h in a facility for the purposes of treatment) for FTH was 76.3% (*n* = 229) and for biomedical care 21.3% (*n* = 64).

**Table 3:** Prevalence of care receipt by type of provider

Care Type		Number	Percent
Inpatient care-Lifetime (n = 300)	Biomedical	64	21.3
	FTH	229	76.3
Inpatient care-Most recent episode (n = 300)	Biomedical	22	7.3
	FTH	114	38.0
Outpatient care-Lifetime (n = 299)	Biomedical	174	58.2
	FTH	254	84.9
Outpatient care- Most recent episode (n = 299)	Biomedical	120	40.1
	FTH	164	45.2
Informal sector (lifetime)			
	Family	286	95.7
	Neighbours	69	23.0
	Religious organisations	31	10.3
	Social groups (Idir)	10	3.3
	NGOs	5	1.7
	Friends (*n* = 292)	46	15.8
	Self-support/self help	257	85.7
	Community support	69	23.0

*FTH* Faith and Traditional Treatment, *NGOs* Non-Governmental Organizations

#### Current access gap

Access to outpatient care for a biomedical psychiatric service provider was 40.1% (*n* = 120) and for that of FTH provider was 54.8% (*n* = 164) corresponding with a current access gap of 59.9% for biomedical care and 45.2% for FTH. A much lower proportion of people reported admission for their current episode either to psychiatric hospitals (n = 22; 7.3%) and/or FTH providers (*n* = 118; 38.0%).

#### Adequacy, quality and equity gaps

Regarding adequacy of biomedical care received in the current episode (Table 4), 31.2% of those who accessed care (*n* = 34/109) reported minimally adequate care. This equates to only 11.3% of the total sample of participants (n = 34/300). The overall satisfaction in care, measuring the presumed construct of quality of care, was generally good, with 68.5% of those using biomedical care reporting satisfaction with the service.

**Table 4:** Adequacy, quality and perceived benefit of care for treatment in recent episode

Service characteristic		Service type			
		Biomedical		FTH	
		N	Percent	N	Percent
Adequacy of care (Biomedical = 109)^a^	Inadequate treatment	75	68.8	–	–
	^b^Minimally Adequate	34	31.2	–	–
Perceived benefit (N=Biomedical = 112) (N=Holy water = 149)	Complete improvement	37	33.0	49	32.9
	Some improvement	63	56.3	76	51.0
	No improvement	12	10.7	23	15.4
	Harm	0	0.0	1	0.7
Satisfaction in care (measuring quality) Biomedical (111) (FTH = 150)	Very satisfied	34	25.5	21	14.0
	Satisfied	46	43.0	52	34.7
	Neutral	21	18.8	32	21.3
	Dissatisfied	7	8.1	32	21.3
	Very dissatisfied	3	4.7	13	8.7

^a^Data not collected for Faith & Traditional providers as there is no guideline for this

*FTH* Faith and Traditional Treatment

^b^Minimally adequate treatment defined as receipt of appropriate treatment with at least four monitoring visits

The perceived benefit and satisfaction measuring quality of care from biomedical care and a specific type of FTH (holy water) was comparable. However, other FTHs, in addition to having been used less, were considered of lower quality and associated with reports of higher harm.

Those with formal education (Adj. OR; 95% CI = 2.1; 1.2, 3.8) and those who had used FTH (Adj. OR; 95% CI = 3.2; 1.9, 5.4) were more likely to use biomedical care (Table 5).

**Table 5:** Associations of selected patient characteristics and likelihood of receiving biomedical treatment in the last 12 months

Characteristics	Response categories	Number interviewed	% who received biomedical treatment	Crude Odds Ratio (95% Confidence Interval)	Adjusted Odds Ratio (95%Confidence Interval)
Sex	Male	172	36.6		Ref
	Female	128	44.5	1.39 (0.87,2.21)	1.55 (0.92, 2.61)
Residence	Urban	60	41.7		Ref
	Rural	239	39.8	0.92 (0.52,1.64)	1.11 (0.57,2.18)
Education	Illiterate	157	32.5		Ref
	Read and write	53	37.7	1.26 (0.66,2.41)	1.27 (0.62, 2.62)
	Formal Education	89	53.9	2.43 (1.43,4.15)	2.40 (1.27,4.53)
Relative wealth	Low or very low	191	38.7		Ref
	Medium or above	109	42.2	1.15 (0.72,1.86)	0.96 (0.57,1.62)
Received traditional treatment in the last 12 months	No	136	25.0		Ref
	Yes	164	52.4	3.31 (2.02,5.42)	3.22 (1.90,5.49)
			Mean (SD)		
Age		300	35.5 (13.5)	0.99 (0.97,1.00)	1.00 (0.98,1.02)
BPRSE		294	48.5 (15.6)	1.00 (0.98,1.01)	1.00 (0.98,1.02)
Social support		300	9.4 (2.4)	1.09 (0.99,1.21)	1.06 (0.95, 1.19)

*BPRSE* Brief Psychiatric Rating Scale Expanded Version

### Potential consequences of the treatment gap

**Fig. 3:**
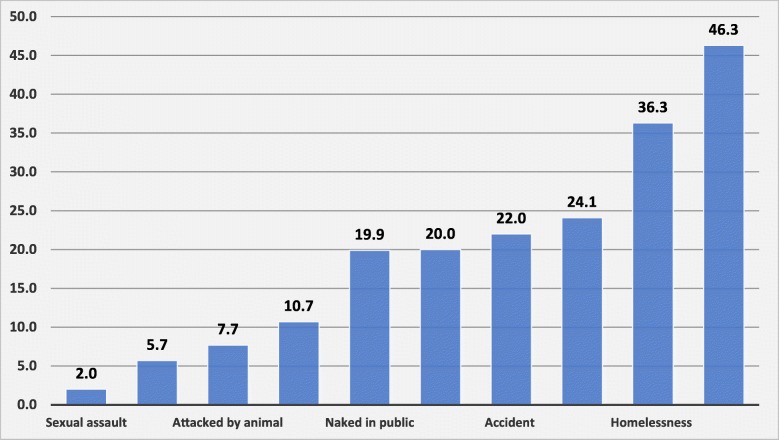
Potential consequences of the treatment gap

Several adverse outcomes and experiences were recorded (Fig. 3) although not all may be accounted for by the treatment gap. The most common were experiences of physical restraint, reported by 46.3% (*n* = 139) of participants. Experience of homelessness also affected more than a third of the sample (36.3%, *n* = 109). Other traumatic experiences included physical assault, sexual assault and accidents. Further exploration of the potential relationship between such adverse outcomes and adequacy of biomedical care suggested a link with not receiving minimally adequate biomedical treatment (See Additional file 1). However, in regression analysis, there was no significant association between the treatment gap and selected adverse outcomes (homelessness, restraint and assault) (Figures not shown).

### Access to other sources of care

The family was reported to be the main source of support for patients, with less than a quarter reporting any input from neighbours (23.0%), friends (15.8%), the community (23.0%), social organisations (3.3%), religious institutions (10.3%) or NGOs (1.7%). On the other hand, almost the same proportion who reported support also reported harm from these resources.

## Discussion

To our knowledge this is the first in-depth exploration of the mental health treatment gap and its potential impact in Africa or any other LMIC setting. Although not observed nationally [9], the study indicates a twofold reduction in the lifetime access gap since the first report of the treatment gap in the neighbouring district of Butajira 15 years earlier (90% vs. 42%) [11, 37]. This difference might have been partly due to the Butajira research project on severe mental disorders that has been operating over the past 15 years and supporting access to biomedical care [12]. Therefore, people in our study site, which is only about 30 kms from Butajira, are more likely to benefit from the service in Butajira. However, key informants, particularly health extension workers, are more likely to recognise those with more severe illness and those who may already be known to the community and on treatment. This can underestimate the treatment gap. Nevertheless, even with the potentially underestimated treatment gap figure, the lifetime treatment gap remains too high and access to minimally adequate care unacceptably low. This study also demonstrates that equity may be an important issue as education and access behaviour were associated with access to biomedical care.

FTHs are the predominant source of care in the study area and more broadly in Ethiopia and will remain important in the longer term. Holy water treatment had good perceived benefit and satisfaction. However, there is no objective evidence that FTHs help in improving severe mental disorders [38] and the self-reported improvement in this study might in part be to do with the religious consonance of the treatment modality, given most patients were Orthodox Christians. Objective investigation of potential benefits and potential synergy with biomedical care is required. Anecdotal experience suggests some of the FTH providers, such as *tenquay* (soothsayer)*,* are less acceptable and their use is likely to be higher than reported. Yet, given the higher rates of reported harms among users of these treatments, further investigation of their use and working with the public to ensure protection of patients is important.

Although families have some role in the care of patients with mental illness globally, the family is the “critical unit” [39] of care in LMICs. Virtually all care in this setting is provided by the family. Despite the availability of a wide range of community resources, including nearly 300 social organisations, over 400 religious groups, NGOs and other resources in the study district [40], access to such community resources was disappointingly low leaving the burden of care almost entirely on the family. Mobilising these resources through additional interventions, for example applying the Basic Needs model [41] or the Community Based Rehabilitation Model that is being employed in an ongoing clinical trial study in the area [42, 43], may be important.

The high level of traumatic experiences such as physical restraint, homelessness and actual physical abuse of people with psychosis is of major concern. Although the traumatic experiences may not entirely be a direct result of the treatment gap, the large treatment gap is likely to be contributory to these negative experiences. In rural villages, people with psychosis induce fear and are perceived as unpredictable and violent [44]. Such a perception, combined with lack of effective treatment, may lead to restraint and even other physical abuse. Preliminary work in the setting indicates that the lack of care alternatives may be the overriding reason for the physical restraint [15]. The lack of legal mechanisms, low awareness among the public about mental disorders and the place of people with mental illness in society exposes people with mental illness to harm.

Scaling up mental healthcare is a crucial step for addressing the broader violation of the rights of people with mental illness [45]. As shown, providing minimally adequate care may reduce these violations and victimisations although the study design would not allow us to confirm this conclusively.

Several limitations to this study are worth mentioning. First, the study is cross-sectional, yet many of the questions ask for lifetime recall. This was unavoidable because part of the focus of the study was intentionally lifetime experience as important index of the level of neglect. Second, although the tool for measuring treatment gap was developed carefully by mental health researchers and practitioners, including social workers, with understanding of the local context, the measure would benefit from further adaptation and simplifying. For example, the measure of the quality of care was assessed through satisfaction in care. Satisfaction is only one dimension of quality of care and evidently inadequate to evaluate quality of care; nevertheless, satisfaction may serve as a simple proxy measure in large population-based studies. Adequacy of care was also measured in a relatively crude way although the measure has been applied previously. We also conducted an analysis of association between adequacy of care for treatment received for the most recent episode and lifetime untoward experiences or abuses. This was carried out as an exploratory examination of the potential impact of the treatment gap. On the other hand, we expected that the pattern of neglect or abuse would be consistent over the course of the illness. If a patient is restrained in one episode, we anticipated that that patient is more likely to be restrained in subsequent episodes unless adequate treatment was provided. The effectiveness and recovery gaps were also not measured because doing so would require prospectively following up participants.

## Conclusion

Viewing the treatment gap in psychosis as a multi-dimensional construct offers a more realistic and holistic understanding of the need for care and may assist policy and advocacy efforts. The community and NGOs can play a bigger role in supporting mental healthcare in rural Ethiopia. Our findings indicate the need to further increase service availability and the need to ensure adequacy of treatment. The use of other FTH is probably higher than reported; this study calls for further robust data on the benefits and harms of FTH and potential synergy with biomedical care. Cultural competence in protecting the dignity of people with mental illness should be a priority for providers and governments.

## Supplementary information

**Additional file 1.** Exploratory analysis of “adequacy” of treatment measured through frequency of visit to biomedical provider and relationship with measures of adverse illness outcomes.

## Data Availability

The data are part of the PRIME project and will be made available at the end of the project. But the data may be requested from the corresponding author for verification of the analyses in this paper.

## Abbreviations

BPRS- E: Brief Psychiatric Rating Scale Expanded version
FTH: Faith and Traditional Healing
ICD: International Classification of Diseases
LMIC: Low and Middle-Income Countries
NGOs: Non-Governmental Organisations
OPCRIT: Operational Criteria for Research
OSS: Oslo 3 Social Support Scale
PRIME: Programme for Improving Mental Healthcare
SCAN: Schedules for Clinical Assessment in Neuropsychiatry
SNNPR: Southern Nations, Nationalities and Peoples’ Region
TGQ: Treatment Gap Questionnaire
WHODAS: World Health Organization Disability Assessment Schedule
