# Supplementary material for: The psychosis treatment gap and its consequences in rural Ethiopia
Source: BMC Psychiatry. 2019 Oct 29;19:325. doi: 10.1186/s12888-019-2281-6 (PMC6819476; doi:10.1186/s12888-019-2281-6)
Supplement: Supplementary file 1 — Additional file 1. Exploratory analysis of “adequacy” of treatment measured through frequency of visit to biomedical provider and relationship with measures of adverse illness outcomes. [file 12888_2019_2281_MOESM1_ESM.docx]

Additional file1. Exploratory analysis of "adequacy" of treatment measured through frequency of visit to biomedical provider and relationship with measures of adverse illness outcomes

|  | **Frequency of treatment** | | | | |  | **Test of difference** | |
| --- | --- | --- | --- | --- | --- | --- | --- | --- |
| **Adverse outcome** |  | Once | 2-3 times | 4-7 times | Over 7 times | Total | X^2^ | P |
| **Homelessness** | No | 37 | 28 | 18 | 17 | 100 | 10.90 | 0.012 |
|  | Yes | 10 | 24 | 4 | 4 | 42 |  |  |
| **Accident** | No | 43 | 36 | 18 | 15 | 112 | 8.20 | 0.042 |
|  | Yes | 4 | 16 | 4 | 6 | 30 |  |  |
| **Beaten** | No | 2 | 7 | 0 | 5 | 14 | 6.81 | 0.078 |
|  | Yes | 2 | 9 | 4 | 1 | 16 |  |  |
| **Assault** | No | 41 | 37 | 18 | 17 | 113 | 4.06 | 0.255 |
|  | Yes | 6 | 15 | 4 | 4 | 29 |  |  |
| **Naked** | No | 42 | 38 | 17 | 20 | 117 | 9.13 | 0.028 |
|  | Yes | 3 | 13 | 5 | 1 | 22 |  |  |
| **Restraint** | No | 28 | 19 | 9 | 12 | 68 | 6.41 | 0.093 |
|  | Yes | 19 | 33 | 13 | 9 | 74 |  |  |
| **Restraint (recent episode)** | No | 10 | 9 | 6 | 4 | 29 |  |  |
|  | Yes | 9 | 24 | 7 | 5 | 45 | 3.78 | 0.287 |
